# Prevalence and correlates of hypothyroidism in pregnancy: a cross-sectional study at Bouget General Hospital, Ivory Coast

**DOI:** 10.11604/pamj.2022.41.37.32553

**Published:** 2022-01-13

**Authors:** Valéry Katché Adoueni, Auguste Jean-Claude Azoh, Ethmonia Kouame, David Guanga Meless, Pascal Sibailly, Augustin Koudou Derbe, Marie-Chantal N´Guessan, Koffi Benjamin Dzade, Simplice Koffi, Théodore Kouakou, Lydie Viviane Arra, Yolande Ouattara

**Affiliations:** 1 Programme National de Lutte contre les Maladies Métaboliques et de Prévention des Maladies non Transmissibles (PNLMM /PMNT), Ministère de la Santé et de l´Hygiène Publique de la République de Côte d´Ivoire (MSHP), Abidjan, Côte d´Ivoire,; 2 UFR Odontostomatologie (UFROS), Université Houphouët Boigny, Abidjan, Côte d´Ivoire,; 3 Institut National de la Santé Publique (INSP), Abidjan, Côte d´Ivoire

**Keywords:** Thyroid disorders, hypothyroidism, Black women, pregnancy

## Abstract

**Introduction:**

several adverse pregnancy outcomes have been reported in gestations with associated hypothyroidism. The prevalence of hypothyroidism has not been frequently reported in Black Africans. This study sorts to report the prevalence and associated factors of hypothyroidism in Black African pregnant women.

**Methods:**

this was a hospital-based cross-sectional study, including all pregnant women attending the gynecologic unit of Bouget General Hospital Abidjan. Serum thyroid-stimulating hormone and T4 were obtained from all participants and analyzed using a fluorescent Immunochemistry assay. Data were analyzed using R version 4.05. Univariable and multivariable logistic regression was used to assess factors associated with hypothyroidism and statistical significance considered as p < 0.05.

**Results:**

overall there were 693 participants, mean age of 28.1(SD 6.4) years with an average gestational age of 24.1 (SD 8) weeks, and a majority of study participants were in the second trimester of gestation. The prevalence of hypothyroidism was 12.1% (n = 84) (10.8% subclinical hypothyroidism and 1.3% clinical hypothyroidism) whereas 1.9% (n = 13) had hyperthyroidism. In addition, patients with reported type 1 diabetes mellitus had an increased risk of hypothyroidism (aOR: 12.6, 95% CI 1.9-100.8; p ≤ 0.01).

**Conclusion:**

this study revealed a high prevalence of hypothyroidism, though mostly in the subclinical form. Further research is warranted to confirm these findings which may have implications on early screening of hypothyroidism in black African women.

## Introduction

Thyroid disorders are of relevance in public health and rank second to diabetes as the most common endocrinological disorder globally [1, 2]. Adverse maternal and fetal outcomes are reported in gestations with associated hypothyroidism [3-5]. It is thus recommended to screen for thyroid disorders during gestation [1, 6]. In addition, pregnancy is marked by several changes including physiological changes in the thyroid gland [7]. The first trimester of pregnancy is accommodated by high levels of human chorionic gonadotropin (HCG) levels which contains a similar alpha (Î±) unit as thyrotropin stimulating hormone (TSH), thus acting on the TSH receptors, leading to a significant decrease in TSH levels during the early phase of pregnancy [7]. However, higher levels of TSH are reported in the later phase of pregnancy due to the progressive decline of HCG levels during gestation [8, 9]. The fetal thyroid gland has been reported to be underproductive in the early half of pregnancy thus there is fetal dependence on adequate levels of maternal thyroid hormones for proper fetal development [5].

The prevalence of hypothyroidism in pregnancy varies across different ethnic groups [10]. In a low to middle-income setting, the prevalence is reported between 5 - 18% [11-13]. Although subclinical hypothyroidism is more prevalent than overt hypothyroidism in pregnancy, studies still report poor neurologic development in children born to mothers with any form of hypothyroidism. In sub-Saharan Africa and specifically the Ivory Coast and to our knowledge, data is scarce on the prevalence of hypothyroidism in pregnancy. Abdulslam *et al*. reported a prevalence of 18% in a group of hypertensive pregnant women in Nigeria [12]. Given the dearth of available literature on hypothyroidism amongst pregnant women in Africa, the study sorts to describe the prevalence and features of hypothyroidism amongst pregnant women in the Ivory Coast.

## Methods

**Study design and study setting:** this was a hospital-based descriptive cross-sectional study conducted from the 6^th^ to the 21^st^ of February 2019 at Port-Bouët General Hospital in Abidjan is the most populated city in the Ivory Coast with a population of 5,355,000 inhabitants. The Port-Bouët General Hospital, located in the commune of the same name in the southeast of the city of Abidjan, is a level 1 reference hospital in the health system pyramid. It receives a population of 75505 people including 13000 pregnant women each year. This hospital provides curative and preventive health care and has several departments, including one dedicated to the consultation of pregnant women. The prenatal consultation service is run by six midwives working under the supervision of gynecologists and obstetricians. Approximately 50 patients receive care here every day.

**Study participants:** eligible study participants were all pregnant women attending the gynecologic unit of the study site. All participants who consented were included in the study. Non-consenting patients were excluded. We calculated a minimum sample size of 55 participants at a 95% confidence interval using the Lorentz formula, assuming a prevalence of hypothyroidism of 16% as reported by Abdulslam *et al*. in Nigeria [12].

**Study variables:** a team of investigators was trained on how to fill in the structured case report form on an electronic tablet. Baseline characteristics including age, parity and gravidity, height, weight, body mass index, and resting blood pressure were collected. A blood sample was drawn from consenting participants and stored. Fluorescent immunochemistry assay mainly Cobas E411 instrument from Roche Diagnostics was used to quantify the level of TSH for all study participants. Thyroid status for all participants was based on the guidelines of the American Thyroid Association (ATA) for diagnosing thyroid diseases in pregnancy [1]. Free T4 levels were assessed for all patients presenting with hypothyroidism.

**Definitions:** forms of hypothyroidism were considered in two stages: subclinical hypothyroidism for TSH between 2.5 and 10 mIU/L and normal free T4 12-22 pmol/L in the first trimester and TSH between 3 and 10 mIU/L and free T4 12-22 pmol/L during the second and third trimester; Clinical hypothyroidism when TSH was between 2.5 and 10mIU/L at first trimester and between 3 and 10mIU/L at the second and third trimester, and free T4 < 12 pmol/L; Hyperthyroidism: TSH < 0.1 mIU/L.

**Statistical analysis:** data were analyzed using R version 4.05, categorical variables are presented as frequency and percentages, and continuous variables as mean and standard deviation. The Chi-squared test was used to compare categorical variables, whereas the independent student T-test was used to compare continuous variables. Univariable and multivariable logistic regression models were used to evaluate patient characteristics associated with hypothyroidism in pregnancy. The selection of variables included in the model was based on current literature on prevalence and risk factors of hypothyroidism in pregnancy. All variables with a p-value of less than 0.2 in the univariable model were included in the multivariable model. Statistical significance was considered as p < 0.05.

**Ethical consideration:** the study protocol was approved by the ethical review boards of the National Ethics and Research Committee of Côte d'Ivoire (CNER-CI) and conducted in compliance with principles laid down in the Declaration of Helsinki and as well as with Côte d'Ivoire´s national ethics principles and regulations. All participants gave their informed consent before joining the study.

## Results

### General characteristics

**Figure 1:**
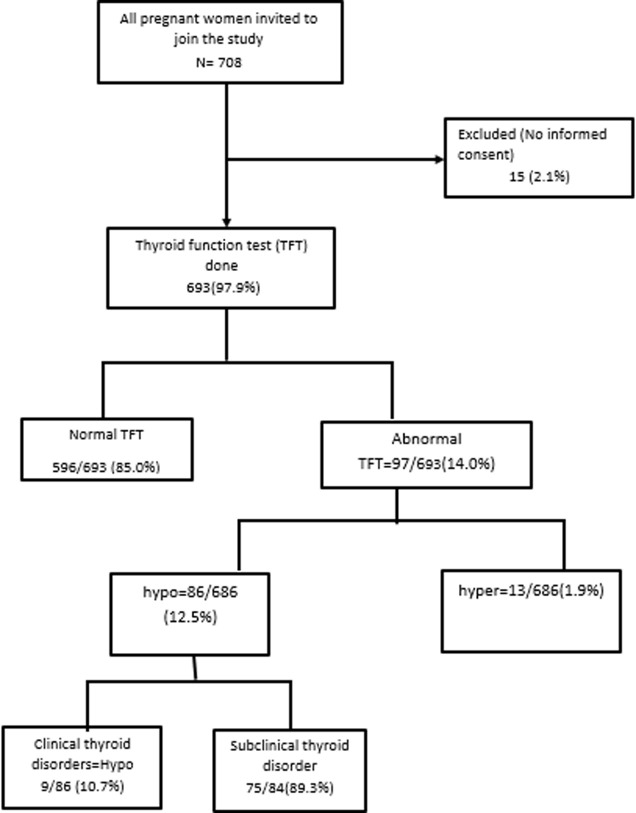
flow chart of the study participants

Of 708 pregnant women eligible for study inclusion, 2.1% (n = 15) were excluded due to refusal to participate (Figure 1). The remaining 693 participants were included in this study. Their age ranged from 12 to 45 years, with a mean age of 28.1 (SD 6.4) years. The mean age was comparable between patients with hypothyroid, euthyroid, and hyperthyroid presentations. The mean gestational age was 24.1 weeks and a majority of the study participants (407/693) were in the second trimester of gestation. More than half of the women were multigravida (55%). Previous miscarriage was found in 30.20% (n = 209) of participants. Also, 7.0% (n = 49) of study participants resided in goiter endemic zones and very few reported current type 1 diabetes mellitus. Table 1 represents the general characteristics of study participants.

**Table 1:** general characteristics of study participants

Maternal characteristic	Mean/frequency	Sd/percentage
Age* (years)	28.1	6.4
Gestational age*	24.1	8.2
BMI* Kg/m^2^	27.4	5.4
Systolic blood pressure* (mmHg)	116.5	14.8
Diastolic blood pressure* (mmHg)	70.6	11.4
Pulse* (beats/minute)	94	11
**Gravidity n**		
Primigravida		23.4
Multigravida	531	76.6
**Parity**		
Nulliparous	208	30.0
Primiparous	199	28.7
Multiparous	286	41.3
**Trimester**		
First	96	13.9
Second	407	58.7
Third	190	27.4
**Miscarriage**		
Yes	209	30.2
No	484	69.8
**Staying in a goitre endemic area**		
Yes	49	7.1
No	644	92.9
**History of thyroid dysfunction**		
Yes	1	0 .1
No	691	99.9
**Known type 1 diabetes**		
Yes	5	0.7
No	687	99.3

*Corresponding mean and standard deviation

### Prevalence and correlates of hypothyroidism in pregnancy

The mean serum TSH was 1.80 (SD 1.02) mIU/L with a linear increase in mean TSH from the first through the third trimester (1.29, 1.81, 2.05 respectively). Of the 693 participants, 84 (12.1%) had hypothyroidism (Figure 1). In addition, subclinical hypothyroidism was the most represented form of hypothyroidism (75/84, 89.3%). There was no statistically significant difference in the presentation of symptoms of hypothyroidism (weight gain, constipation, cold intolerance, muscle cramps) between patients with or without hypothyroidism. Also, there was a slight increase in crude odds (OR: 1.3, 95% CI 0.7-2.6; p = 0.5) of hypothyroidism amongst participants in the third trimester of gestation though not significant. After adjusting for age and period of gestation, patients with a history of type 1 diabetes mellitus had an increased association with hypothyroidism (aOR: 12.6, 95% CI 1.9-100.8; p = < 0.01) (Table 2).

**Table 2:** factors associated with hypothyroidism

	Odds ratio	P-value	aOR	p-value
Excess Weight gain	0.8(0.5-1.3)	0.5		
Constipation	1.3(0.8-2.0)	0.3		
Cold intolerance	0.8(0.4-1.5)	0.5		
Muscle cramps	0.8(0.5-1.3)			
Multiparity	0.98(0.6-1.6	0.9		
Multigravida	1.1(0.6-1.9)			
History of abortions	1.3(0.8-2.0)	0.4		
History of type 1 diabetes	11.2(1.8-86.2)	<0.01	12.6(1.9-100.8)	<0.01
**Trimester**				
Second	0.6(0.3-1.1)	0.1	0.6(0.3-1.2)	0.1
Third	1.3(0.7-2.6)	0.5	1.4(0.7-2.8)	0.4
Systolic Bp >= 140 mmHg	0.7(0.2-1.9)	0.6		
Pulse (5-unit change) beats/minutes)	0.9(0.8-1.1	0.5		
BMI ≥ 30 kg/m2	1.2(0.7-1.9)	0.5		

## Discussion

Several adverse outcomes have been described in pregnancies with associated hypothyroidism amongst which poor fetal neurological development [4, 14]. In this study, we aimed to determine the prevalence and associated factors of hypothyroidism in pregnancy. The prevalence of hypothyroidism in this study was 12.1% and there was a strong association with hypothyroidism in participants with concomitant type 1 diabetes mellitus. These results portray the burden of hypothyroidism in pregnancy and highlight the importance of further research, which may have some implications, including an early screening of hypothyroidism in pregnancy in an African context to prevent poor maternal and fetal outcomes during pregnancy and after delivery.

The prevalence of hypothyroidism in our study population is similar to the reports from other low to middle-income settings [13, 15-17]. A higher prevalence (16%) was however reported in Nigeria by Abdulslam K *et al*. [12]. This difference in prevalence might be explained by different populations and setting as well as the difference in TSH cutoffs. But it should be noted that more than half of the participants in the Nigerian study had associated hypertension contrary to 11% of participants in this study with systolic blood pressure above 140 mmHg. Contrarily, a study in India reported a lower prevalence (6.3%) of hypothyroidism [18]. This discrepancy is explained by the higher trimester-dependent cutoffs of TSH (at least 5IU/ml) used in the Indian study. On the contrary, a lower prevalence of hypothyroidism in pregnancy has been reported in high-income settings [19, 20] which is explained by differences in cutoff values used for diagnosis of hypothyroidism and secondly by the geographical differences in presentation of thyroid diseases [10]. In our study, there was no statistically significant difference in symptoms between those with or without hypothyroidism. Similar findings have been reported by Goel P *et al*. in India [18]. However, it should be noted that thyroid disorders can be hard to diagnose more precisely in pregnancy due to physiological rising levels of TSH but also because pregnancy-related symptoms due to high human chorionic gonadotropin and estrogen can overlap with symptoms of hyperthyroidism.

In our study, multivariable analysis showed that patients with a reported history of type 1 diabetes mellitus had an increased risk of hypothyroidism. This finding has been previously reported by Shahbazian *et al*. who demonstrated in their study that the risk of thyroid dysfunction was more common in participants with pre-gestational or gestational diabetes [21]. Potential explanations include the fact that type 1 diabetes is an autoimmune disorder, patients with type 1 diabetes mellitus could also present with concomitant or cross-reacting autoimmune activity in other endocrine glands including the thyroid [22]. Although the sample for our study was non randomly selected women attending a reference hospital and therefore not nationally representative of African Black pregnant women, and which may therefore present some biases, the potential beneficial impact of this study as first published data cannot be overemphasized. Our findings will serve as a base to design further studies in other settings to better precise the burden of thyroid disorders in African pregnant Black women.

## Conclusion

This study revealed a higher prevalence of hypothyroidism in Black African pregnant women compared to that reported in Caucasians. These results raise awareness of the increased burden of this endocrine disorder and thus substantiate existing literature on the importance of regular screening of thyroid disorders in pregnancy in Africa, consequently avoiding certain adverse pregnancy outcomes.

### What is known about this topic

- *Adverse maternal and fetal outcomes are reported in gestations with associated hypothyroidism;*
- *Hypothyroidism might be prevalent in African pregnant women but has not been well established*.

### What this study adds

- *The prevalence of hypothyroidism in African pregnant women is high, up to 12.1%;*
- *Most of these African pregnant women with hypothyroidism present with subclinical forms of the disease;*
- *Screening of hypothyroidism in Black African women might be relevant to reduce associated adverse maternal and fetal outcomes*.
